# Immunization with outer membrane vesicles drived *Proteus mirabilis p*rotects mice against bacteria-induced lethality

**DOI:** 10.3389/fimmu.2025.1688837

**Published:** 2025-11-11

**Authors:** Wei Fan, Yilu Zhai, Xinyu Zhang, Fuliang Sun, Lin Kong, Wei Wang, Dazhuo Zhao, Jiaqi Fu

**Affiliations:** 1 Department of Veterinary Medicine, College of Agricultural, Yanbian University, Yanji, China; 2 Institute of Special Economic Animal and Plant Sciences, Chinese Academy of Agricultural Sciences, Changchun, China; 3 Department of Animal Disease Prevention and Control Centre, Yanji, China

**Keywords:** outer membrane vesicles, *Proteus mirabilis*, T cells, B cells, vaccine, immuneprotection

## Abstract

**Introduction:**

*Proteus mirabilis* (*Pm*) has emerged as a significant and widespread opportunistic pathogen affecting both humans and animals, resulting in substantial economic losses within the agricultural sector. While most conventional antibiotics remain effective against Pm infections, the rise of multidrug-resistant strains has considerably complicated disease management. Outer membrane vesicles (OMVs), which are vesicular structures secreted by Gram-negative bacteria, have been identified in numerous studies as potential antigenic components or immune adjuvants for the development of novel vaccines.

**Methods:**

In this study, *Proteus mirabilis* outer membrane vesicles (*Pm*_OMVs) were employed to immunize mice, followed by the execution of *Pm* infection assays. The immune responses elicited by *Pm*_OMVs and their protective effects in the Pm infection mice were evaluated using quantitative PCR, ELISA, and Western blot analyses.

**Results:**

Our findings demonstrated that *Pm*_OMVs stimulated B cells to produce specific antibodies and induced Th1/Th17-mediated immune responses. Following 24 hours of *Pm* infection, Th1 and Th17 cells in the *Pm*_OMVs-infected group were activated, releasing substantial levels of cytokines that facilitated rapid bactericidal activity. After 72hours of *Pm* infection, Th2 and Treg cells in this group were activated to suppress excessive inflammatory response and achieve tissue repair. *Pm*_OMVs also specificall increased the survival rate of mice infected with *Pm*, up to 80%.

**Discussion:**

These results suggest that *Pm*_OMVs can be used as an effective material to prevent *Pm* infection.

## 1 Introduction

*Proteus mirabilis*(*Pm*) is a zoonotic conditionally pathogenic bacterium that is highly pathogenic and widespread in nature. It produces several virulence factors during infection, of which the most widely known are adhesins, endotoxins, and flagellins. They cause gastric mucosal and urinary tract damage and severe induction of bacteraemia leading to death in humans and animals (1–3). Currently, the uncontrolled use of antibiotics in large-scale animal husbandry has led to an increasing problem of drug resistance (4). Multidrug-resistant *Pm* has brought great difficulties in clinical medication. This hinders the normal development of the farming industry (5). Meanwhile, the overlapping spectrum of antimicrobial drugs used in humans and animals (6) poses a potential threat to public health safety. Therefore, the development of novel therapeutic alternatives to antibiotics has become crucial to address the problem of drug resistance.

Vaccination is widely regarded as the most cost-effective strategy for the prevention of infectious diseases. Upon exposure to pathogens in vaccine-immunized mice, dendritic cells promptly identify pathogenic components and subsequently activate B and T lymphocytes, leading to the production of specific antibodies and cytokines that initiate adaptive immune responses (7). Both B and T cells are integral to the host defense against bacterial infections (8). The B-cell-mediated immune response generates specific antibodies, including IgG, IgM, and IgE, which facilitate macrophage-mediated phagocytosis and activate the complement cascade, culminating in the formation of the membrane attack complex (MAC) that compromises bacterial cell integrity (9). In the context of diverse bacterial infections, the appropriate differentiation of T helper (Th) cells is critical for an effective T-cell immune response (10). For instance, Th1 cells secrete cytokines that enhance macrophage phagocytic activity and promote natural killer (NK) cell-mediated cytotoxicity (11). Conversely, Th2 cells are implicated in anti-inflammatory processes and tissue repair, providing defense against pathogenic bacteria invading the gastrointestinal tract (12). Additionally, Th17 cells recruit neutrophils to infection sites to eliminate extracellular bacteria and fungi (13). Beyond Th cells, regulatory Treg cells play a pivotal role in suppressing excessive inflammatory responses, thereby preventing inflammatory storms that can cause tissue damage (14). Notably, conventional vaccines cannot often effectively activate both B and T cell responses (15). Consequently, there is a pressing need to develop novel, complex vaccines that exhibit robust safety profiles while eliciting comprehensive protective immunity.

Outer membrane vesicles(OMVs) are lipid bilayer vesicles secreted by Gram-negative bacteria with a diameter of approximately 100–400 nm. They consist of lipopolysaccharides (LPSs), outer membrane proteins (OMPs), and inclusions (virulence factors, nucleic acids, and enzymes) (16). Most of the biomolecules secreted by pathogen-derived OMVs are associated with invasion, adhesion, host cell damage, immunomodulation, and virulence enhancement (17). It has been shown that OMVs secreted by Neisseria meningitidis (18), Pseudomonas aeruginosa (19) and Helicobacter pylori (20) protect infected animal models. Compared to traditional bacterial vaccines, OMVs exhibit advantages such as high immunological safety and biological inertness, and they are non-replicative when employed as immunological agents (21). Furthermore, OMVs harbor multiple pathogen-associated molecular patterns (PAMPs) that stimulate the immune system and present multivalent antigens capable of inducing both B-cell activation and T-cell differentiation, thereby eliciting broader protective immunity (22). This immune response is characterized by its long-term stability and specificity, effectively targeting pathogenic organisms while sparing healthy cells. Consequently, OMVs represent promising candidates for antigen development in vaccine research.

To date, relatively few studies have been conducted on *Pm*_OMVs, especially in terms of immunoprotection. Consequently, the present study aimed to elucidate the immunoprotective properties and underlying mechanisms of *Pm*_OMVs by establishing both a *Pm*_OMVs-immunized model and a *Pm*_OMVs immunoinfection model. The findings of this investigation are intended to provide a theoretical foundation for the development of *Pm*_OMVs vaccines.

## 2 Materials and methods

### 2.1 Ethical certification

All the animal care and use programs were performed according to the Regulations on the Management of Laboratory Animals approved by the State Council of the People’s Republic of China. All the animal experiments were approved by the Animal Ethics and Experimentation Committee of Yanbian University (Jilin, China, Ethics Approval No. YD20240122001) by its regulations.

### 2.2 Bacterial strains and ultrafiltration precipitation method for the preparation of *Pm*_OMVs

*Pm* was cultured in 1 L of Luria-Bertani (LB) medium at 37°C with agitation at 200 rpm until the optical density at 600 nm (OD600) reached approximately 1.2. The supernatant was then harvested by centrifugation at 8,000 × g for 30 minutes and was filtered through a 0.22 μm membrane filter (Millipore Corporation, Bedford, MA) to remove residual cells. Subsequently, the filtrate was concentrated to a volume of 5 mL using an ultrafiltration device (Beyotime Corporation, Shanghai, China) with a molecular weight cutoff of 50 kDa. The concentrate was combined with an equal volume of polyethylene glycol (PEG) 10,000 solution (Beyotime) and stored at 4°C until complete precipitation occurred. The precipitated material was then resuspended in 1 mL of phosphate-buffered saline (PBS) at pH approximately 7.4 and stored at -80°C for further use.

### 2.3 Characterization and analysis of OMVs

To investigate the ultrastructural features of OMVs, a 10 μL aliquot of the OMVs suspension was applied onto a copper grid. The sample was then incubated at ambient temperature for 10 minutes, followed by a brief rinse with distilled water for 30 seconds. After removing excess fluid, the grid was subjected to negative staining by the dropwise addition of 2% uranyl acetate dihydrate (Yaji Biological Corporation, Shanghai, China), followed by the application of 10 μL hydrogen peroxide acetate for one minute. Excess stain was absorbed using filter paper, and the OMVs were air-dried under an incandescent lamp for two minutes before examination via transmission electron microscopy. For particle size analysis, the OMVs samples were diluted 1:50 and assessed using a nanoparticle tracking analyzer. Protein concentration was quantified employing the Bradford assay (Servicebio, Wuhan, China). Additionally, protein separation and compositional analysis were conducted through 10% sodium dodecyl sulfate-polyacrylamide gel electrophoresis (SDS-PAGE) (Servicebio) and Coomassie brilliant blue staining.

### 2.4 Mouse immunity and infection

Immunization Protocol Using *Pm*_OMVs in Mice: Twelve six-week-old BALB/c mice were randomly divided into two groups: a naive control group (NC) and a group immunized with Pm_OMVs. The control group received intraperitoneal injections of 0.2 mL PBS, while the immunized group was administered 0.2 mL of Pm_OMVs at a concentration of 400 μg/mL. Both groups received identical doses on days 7, 14, and 21 following the initial immunization. On day 28 post-primary immunization, blood samples were collected via tail vein puncture from each mouse, and serum was isolated. Subsequently, the animals were euthanized by CO2 inhalation, and the spleens were harvested. A second cohort of twelve six-week-old BALB/c mice underwent the same immunization schedule. At 42 days post-initial immunization, these mice were euthanized by CO2 inhalation, and serum as well as heart, liver, spleen, lung, and kidney tissues were collected for further analysis.

Sub-lethal *Pm*-infected mice: Eighteen six-week-old BALB/c mice were randomly divided into three groups: naive control group (NC), *Pm*-infected (*Pm*), and *Pm*_OMVs immunoinfection (*Pm*_OMVs-infected). The NC group was injected intraperitoneally with 0.2 mL PBS at 42 days after the first immunization, and the remaining two groups were injected intraperitoneally with 0.2 mL of a sublethal dose(3×10^6^ CFU) of *Pm*. Lungs, livers, and spleens were collected 24 h after infection. Eighteen 6-week-old BALB/c mice were again selected, grouped, immunized, and infected according to the above procedure. Lungs, livers, and spleens were collected at 72 h post-infection for subsequent experiments.

### 2.5 *Pm*_OMVs endotoxin content detection

The endotoxin content of *Pm*_OMVs was measured following the protocol provided by the endotoxin assay kit (Beyotime).

### 2.6 Mice body weight and organ coefficients

The body weight of each group of mice was measured at 0, 7, 14, 21, 28, and 42 days following immunization with *Pm*_OMVs. Additionally, the weights of organs were recorded at 42 days post-immunization. The organ index was calculated using the formula: organ coefficient = (organ weight (g)/mouse body weight (g)) × 100%.

### 2.7 Bacterial load

To evaluate the bacterial load in the liver and lungs of each group, samples were collected at 24 and 72 hours after *Pm* infection. Specifically, 0.1 g of lung and liver tissues were weighed and homogenized, and bacterial loads were quantified using the colony counting method.

### 2.8 Physiological and biochemical indicators

Total serum protein, aspartate aminotransferase, urea nitrogen and other physiological and biochemical indexes were measured in serum of each group by automatic physiological and biochemical analyzer.

### 2.9 RNA extraction and qRT-PCR

Total RNA was isolated from the spleen tissues of each experimental group following the protocol provided with the Total RNA Extraction Kit (Beyotime). Subsequently, first-strand cDNA synthesis was performed utilizing the gDNA Eraser reagent (Sangon Biotech, China). Quantitative real-time PCR (qRT-PCR) was conducted to assess the expression levels of IFN-γ, IL-4, IL-6, IL-17, TGF-β, GATA-3, T-bet, RORγt, and FoxP3 genes, using the synthesized cDNA as the template. GAPDH served as the endogenous reference gene, and relative gene expression was calculated employing the 2^−ΔΔCT^ method. Detailed sequences of gene-specific primers are provided in **Supplementary Material S1**.

### 2.10 HE and Masson

Fresh tissue specimens from each experimental group were collected and fixed in a 10% formaldehyde solution for a duration of 48 hours. After fixation, the samples were dehydrated by an ethanol series graded and then cleared with xylene. Subsequently, the tissues were embedded in paraffin and cut into sections of 4 µm thickness. Tissue staining was performed using hematoxylin and eosin (HE) as well as Masson’s Trichrome Staining Kit (Solarbio, China) according to the manufacturer’s instructions. Stained sections were analyzed under a light microscope at 200 × and 400× magnification. Each pathological specimen was assessed on a scale of 0 to 5, with 0 indicating no abnormality and 5 indicating the most severe condition. This assessment was based on established criteria, including hyperemia, edema, hemorrhage, and neutrophilic infiltration (23).

### 2.11 Detection of specific antibodies and cytokines in mice immunized with *Pm*_OMVs

Serum was collected from each group and analyzed using ELISA. To detect specific IgG, IgG1, IgG2a, IgE antibodies, microtiter plate wells were coated with *Pm*_OMVs at a concentration of 0.4 mg per well and incubated overnight at 4°C. Serum samples were diluted 1:1000 in PBS and applied as primary antibodies. Enzyme-conjugated goat anti-mouse secondary antibodies specific for IgG, IgG1, or IgG2a (MEIMIAN, JiangSu, China) were employed. All assays were conducted in accordance with the manufacturer’s guidelines, and absorbance measurements were taken at 450 nm (OD450). Cytokine levels, including TNF-α, IFN-γ, IL-4, IL-6, IL-10, T-bet, GATA-3, TGF-β, IL-17A, were quantified using ELISA following the protocols provided by the manufacturer (MEIMIAN).

### 2.12 Western blot

Protein extracts were resolved by SDS-polyacrylamide gel electrophoresis under reducing conditions using a 12% gel. Subsequently, the proteins were transferred onto PVDF membranes in a Tris-glycine fast transfer buffer at a constant current of 300 mA for 1 hour. The membranes were then blocked with a rapid blocking solution for 1 hour, followed by an overnight incubation at 4 °C with the primary antibody diluted at 1:1000 (UpingBio, China). After washing, the membranes were incubated with horseradish peroxidase (HRP)-conjugated goat anti-rabbit IgG secondary antibody (1:5000, Proteintech Group, China). Protein bands were visualized using an enhanced chemiluminescence (ECL) detection system. Densitometric analysis of the bands was conducted using ImageJ software.

### 2.13 Immunoprotection

This study aimed to evaluate the protective effect of *Pm*_OMVs in mice infected with *Pm*. Thirty six-week-old BALB/c mice were randomly assigned to three groups: a naïve control group(NC), *Pm*-infected group (*Pm*), and *Pm*_OMVs-immunoinfection group (*Pm*_OMVs-infected). Following immunization with *Pm*_OMVs and subsequent *Pm* infection according to established protocols, the survival of mice in each group was monitored and recorded over 7 days.

To assess the specificity of the immune protection conferred by *Pm*_OMVs, mice were grouped and immunized with *Pm*_OMVs following the previously described protocol. Subsequently, a sublethal intraperitoneal dose of 2.4×10^6^ CFU of *Klebsiella pneumoniae* (*KP*) was administered. The survival rates of each group were monitored and documented over 7 days post-infection.

### 2.14 Statistics and analysis

Data analysis was conducted using GraphPad Prism 10 (GraphPad, San Diego, CA) and results are presented as mean ± standard deviation (mean ± SD). Group differences were assessed using one-way or two-way analysis of variance (ANOVA) as appropriate. For comparisons between two groups, the t-test was employed. All experiments were performed a minimum of three times (n = 3), unless otherwise specified. The P<0.05 was considered indicative of statistical significance and ns denotes no significance.

## 3 Results

### 3.1 Characterization of outer membrane vesicles

**Figure 1:**
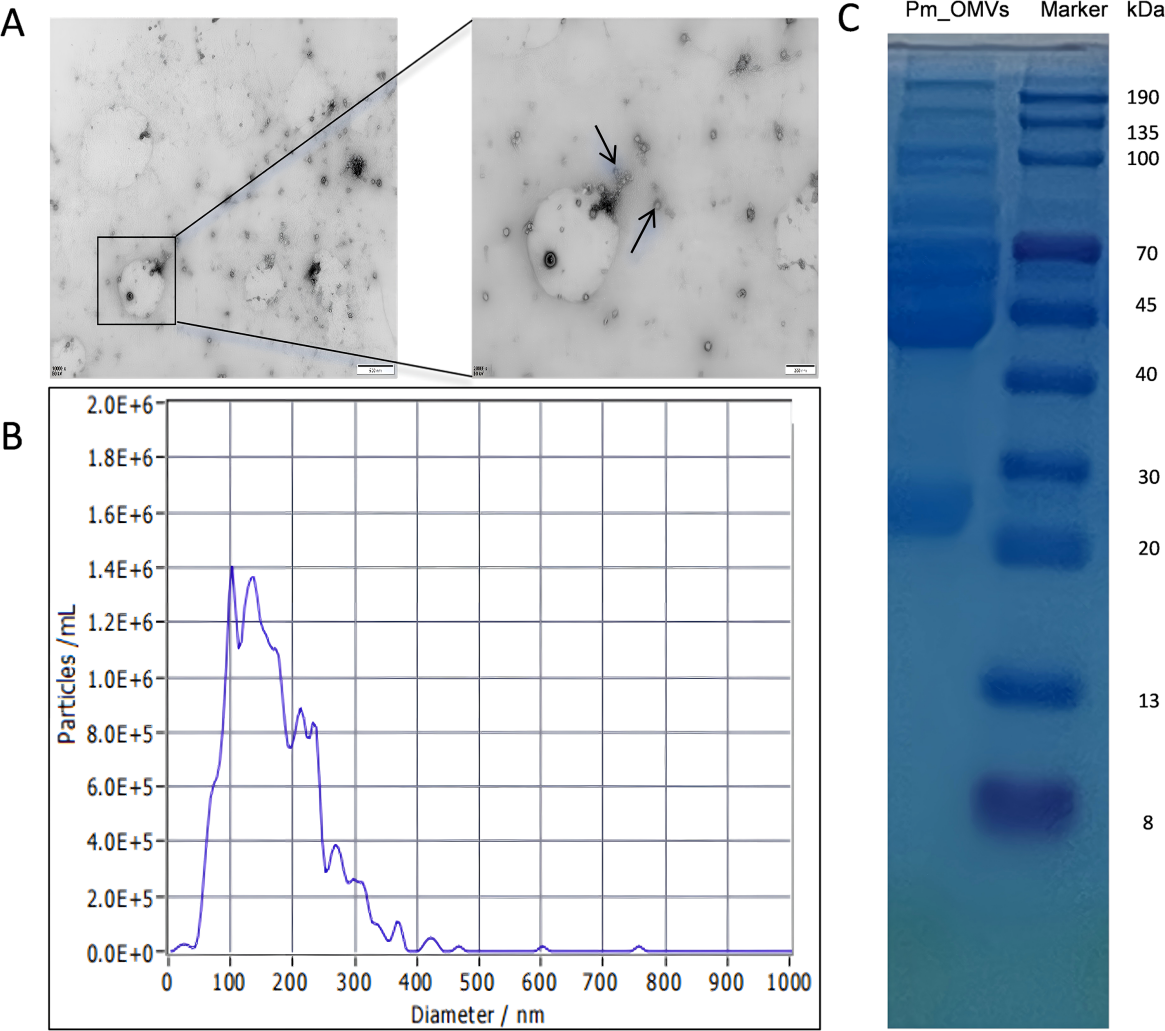
Characterization of outer membrane vesicles of Proteus mirabilis. **(A)** Electron microscopic observation of *Pm*_OMVs at 200 and 500 nm, respectively. **(B)** Particle size distribution of *Pm*_OMVs in the bacterial culture solution. **(C)** Results of Coomassie blue staining of *Pm*_OMVs.

To characterize the *Pm*_OMVs isolated via ultrafiltration precipitation, PBS was employed to collect the final precipitates. Transmission electron microscopy (TEM) analysis revealed numerous transparent, nearly spherical vesicles (**Figure 1A**). Nanoparticle tracking analysis indicated that the size distribution of the outer membrane vesicles predominantly ranged from 100 to 300 nm (**Figure 1B**). To investigate the protein composition of *Pm*_OMVs, 10 μg of the sample was analyzed by 10% SDS-PAGE, which revealed two prominent protein bands at approximately 45 and 70 kDa (**Figure 1C**). The principal outer membrane proteins of *Pm*, namely OmPA and OmpC, exhibit molecular weights in the range of approximately 38 to 45 kDa, while UreC, the large subunit of urease, is observed at approximately 60 to 76 kDa. These findings indicate that the Pm_OMVs isolated retain outer membrane proteins that are essentially consistent with those of the parental bacterial strain. Furthermore, employing the ultrafiltration precipitation technique, approximately 4.08 mg of Pm_OMVs protein was successfully extracted from one liter of *Pm* culture broth, as quantified by the BCA assay.

### 3.2 Safety of *Pm*_OMVs immunization

**Figure 2:**
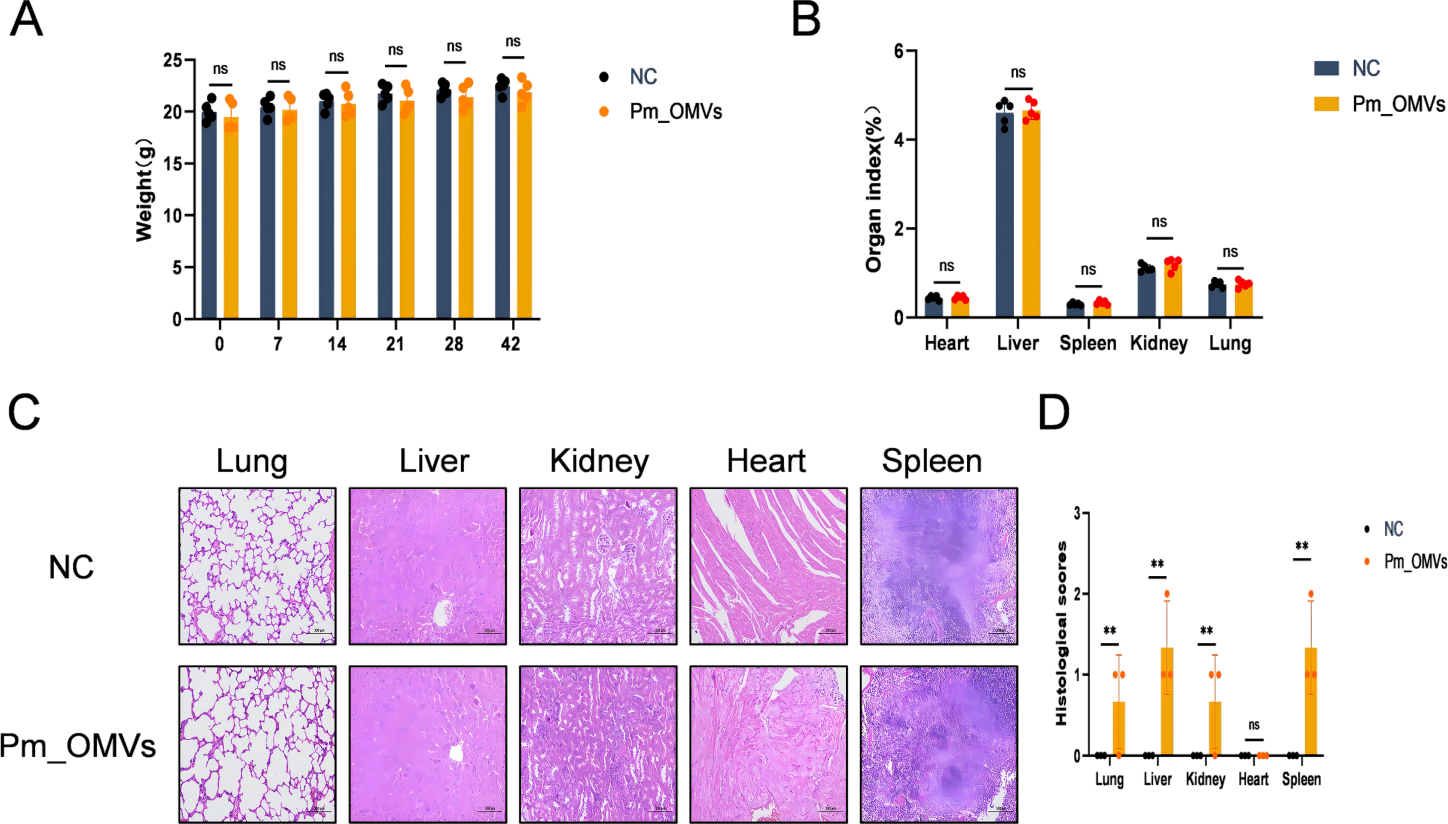
*Pm*_OMVs immunoprotective assessment.**(A)** Effect of *Pm*_OMVs on body weight of immunised mice (n=5). **(B)** Effects of *Pm*_OMVs on organs of immunised mice (n=5). **(C)** Pathological results of NC and *Pm*_OMVs groups (400×) (n=3). **(D)** Pathohistological scoring of *Pm*_OMVs immunised mice (n=3). ^**^P<0.01 refer to the level of significance and ns denotes no significance.

This study aimed to evaluate the safety profile of *Pm*_OMVs immunization. Initially, the endotoxin content of *Pm*_OMVs was quantified, revealing an endotoxin concentration of 1.58×10^4^ EU/mL. After successive steps of membrane filtration, centrifugation, and rinsing with PBS, the endotoxin concentration of *Pm*_OMVs was quantified to 3 × 10^3^ EU/mL. This preparation was used in mouse immunoassays. Subsequently, the body weights of mice in both the NC group and the *Pm*_OMVs group were measured on days 0, 7, 14, 21, 28, and 42 following the initial immunization. Statistical analysis indicated no significant differences in body weight between the *Pm*_OMVs group and the NC group (P > 0.05) (**Figures 2A, B**). Additionally, organ coefficients, histopathological assessments using HE staining, and histological scoring of major organs were performed 42 days following immunization. Results demonstrated no significant differences in organ coefficients between the two groups (P > 0.05). And histological scoring of major organs revealed that, in comparison to the NC group, the *Pm*_OMVs group exhibited significant differences(P<0.01). Analysis of the HE staining results suggests that the observed difference may be attributed to inflammatory cell infiltration rather than underlying pathological changes (**Figures 2C, D**).

To further investigate the potential effects of *Pm*_OMVs on immunized mice, key physiological and biochemical indices were assessed in both the NC group and the *Pm*_OMVs group using a fully automated physiological and biochemical analyzer following 42 days post-immunization (**Table 1**). The results indicated no statistically significant differences in these parameters between the *Pm*_OMVs group and the NC group (P > 0.05). In summary, the administration of *Pm*_OMVs in this study did not elicit any adverse effects in the immunized mice.

**Table 1:** Physiological and biochemical indicators tests in Control, *Pm*_OMVs, *Pm*, and *Pm*_OMVs-infected mice.

Indicators	Reference range	*Pm*_OMVs	*Pm*_OMVs-infected	*Pm*	NC
Serum total protein(g/L)	36.00-66.00	59.80 ± 1.35	53.00 ± 0.42	52.0 ± 2.64	56.90 ± 3.52
Alanine aminotransferase(U/L)	40.3-47.06	25.70 ± 0.68	49.02 ± 1.98^**^	82.58 ± 4.92	18.90 ± 2.05
Aspartate aminotransferase(U/L)	59.00-247.00	251.00 ± 0.51	254.00 ± 1.15^**^	540.00 ± 15.26	176.00 ± 13.61
Creatinine(mol/L)	12.0-71.0	12.40 ± 0.29	17.2 ± 3.51	8.65 ± 1.03	15.60 ± 2.94
Uric acid(mol/L)	101.00-321.00	157.92 ± 8.48	91.88 ± 15.7	224.32 ± 12.58	132.10 ± 13.26
Urea nitrogen(mmol/L)	4.00-11.80	9.47 ± 1.37	7.71 ± 0.82^**^	15.34 ± 1.67	10.00 ± 2.01

^**^P<0.01 refer to the level of significance.

### 3.3 *Pm*_OMVs induces Th1/Th17 dominant differentiation

**Figure 3:**
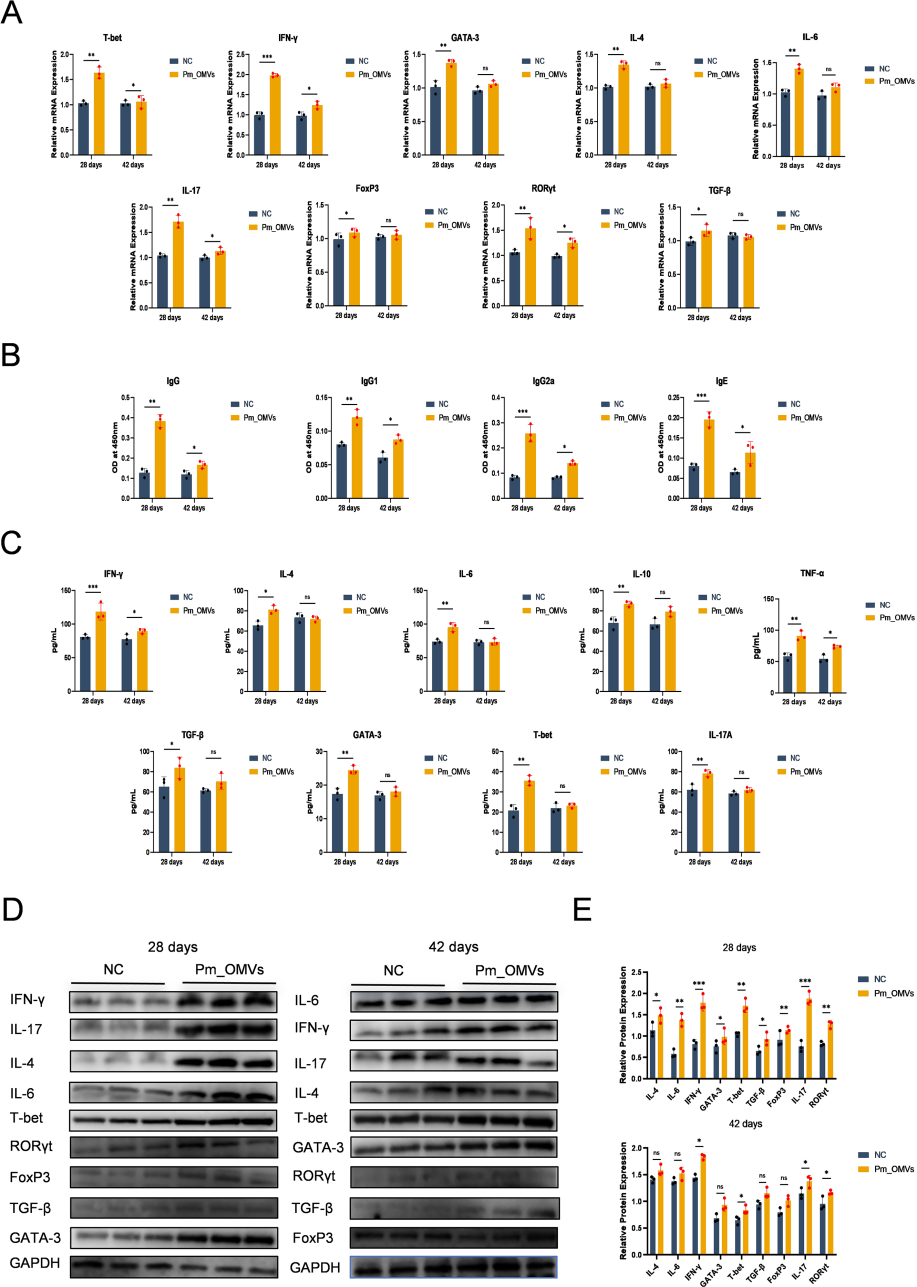
Effects of *Pm*_OMVs on Th cell differentiation in mice **(A)** Effects of *Pm*_OMVs on IFN-γ, IL-4, IL-6, IL-17, TGF-β, GATA-3, T-bet, RORγt, and FoxP3 mRNA expression levels in immunised mice (n=3). **(B)** Effect of *Pm*_OMVs on the secretion levels of specific antibodies IgG, IgG1, IgG2a, and IgGE in immunised mice (n=3). **(C)** Effects of *Pm*_OMVs on the secretion levels of cytokines IFN-γ, IL-4, IL-6, IL-17A, TGF-β, GATA-3, T-bet, and IL-10 in immunised mice (n=3). **(D, E)** Effects of *Pm*_OMVs on protein expression levels of IFN-γ, IL-4, IL-6, IL-17, TGF-β, GATA-3, T-bet, RORγt, and FoxP3 in immunised mice (n=3). ^***^P<0.001, ^**^P<0.01, ^*^P<0.05 refer to the level of significance and ns denotes no significance.

To investigate the immune response induced by *Pm*_OMVs, an *in vivo* immunological model of *Pm*_OMVs was established in this study. As shown in **Figure 3A**, compared with the NC group, 28 days after the first immunization with *Pm*_OMVs, the expression levels of IFN-γ, IL-4, IL-6, IL-17, TGF-β, GATA-3, T-bet, RORγt, and FoxP3 genes in the *Pm*_OMVs group were significantly increased (P < 0.01). In contrast, 42d after the first immunization with *Pm*_OMVs, there was no significant difference in the expression levels of the genes in the *Pm*_OMVs group compared with the NC group, except for the expression levels of the genes IFN-γ, IL-4, IL-17, T-bet, RORγt, and FoxP3, which were significantly higher (P < 0.05). In addition, the specific antibody ELISA results (**Figure 3B**) showed that compared with the NC group, the secretion levels of specific antibodies IgG, IgG1, IgG2a, and IgE were significantly increased in the *Pm*_OMVs group 28, 42 d after the first immunization (P<0.01). This suggests that *Pm*_OMVs can induce long-term and stable expression of specific antibodies.

To further elucidate the immune response pattern elicited by *Pm*_OMVs, sera were collected from mice in both the NC and *Pm*_OMVs groups at 28 and 42 days following the initial immunization with *Pm*_OMVs. Cytokine levels were quantified using an ELISA kit. As illustrated in **Figure 3C**, at 28 days post-immunization, the *Pm*_OMVs group exhibited significantly elevated secretion of cytokines, including IFN-γ, IL-4, IL-6, IL-17A, TGF-β, GATA-3, T-bet, and IL-10 compared to the NC group (P < 0.01). However, by 42 days post-immunization, no significant differences in cytokine secretion were observed between the *Pm*_OMVs and control groups (P > 0.05). Complementary Western blot analyses (**Figures 3D, E**) demonstrated that at 28 days post-immunization, protein expression levels of IFN-γ, IL-4, IL-6, IL-17, TGF-β, GATA-3, T-bet, RORγt, and FoxP3 were markedly higher in the *Pm*_OMVs group relative to the NC group (P < 0.01). At 42 days post-immunization, significant increases in the expression of T-bet, RORγt, IFN-γ, and IL-17 proteins persisted in the *Pm*_OMVs group compared to controls (P < 0.05), whereas the expression levels of the remaining proteins did not differ significantly.

Integrating the findings from the experiments, the secretion levels of cytokines, as well as the gene and protein expression of differentiation markers associated with Th1 and Th17 cells, were elevated compared to those of Th2 and Treg cells at 28 and 42 days following the initial immunization with *Pm*_OMVs. These results suggest that *Pm*_OMVs predominantly elicit a Th1/Th17-skewed immune response.

### 3.4 *Pm*_OMVs protects *Pm*-infected mice by inducing Th cell polarization pathway

**Figure 4:**
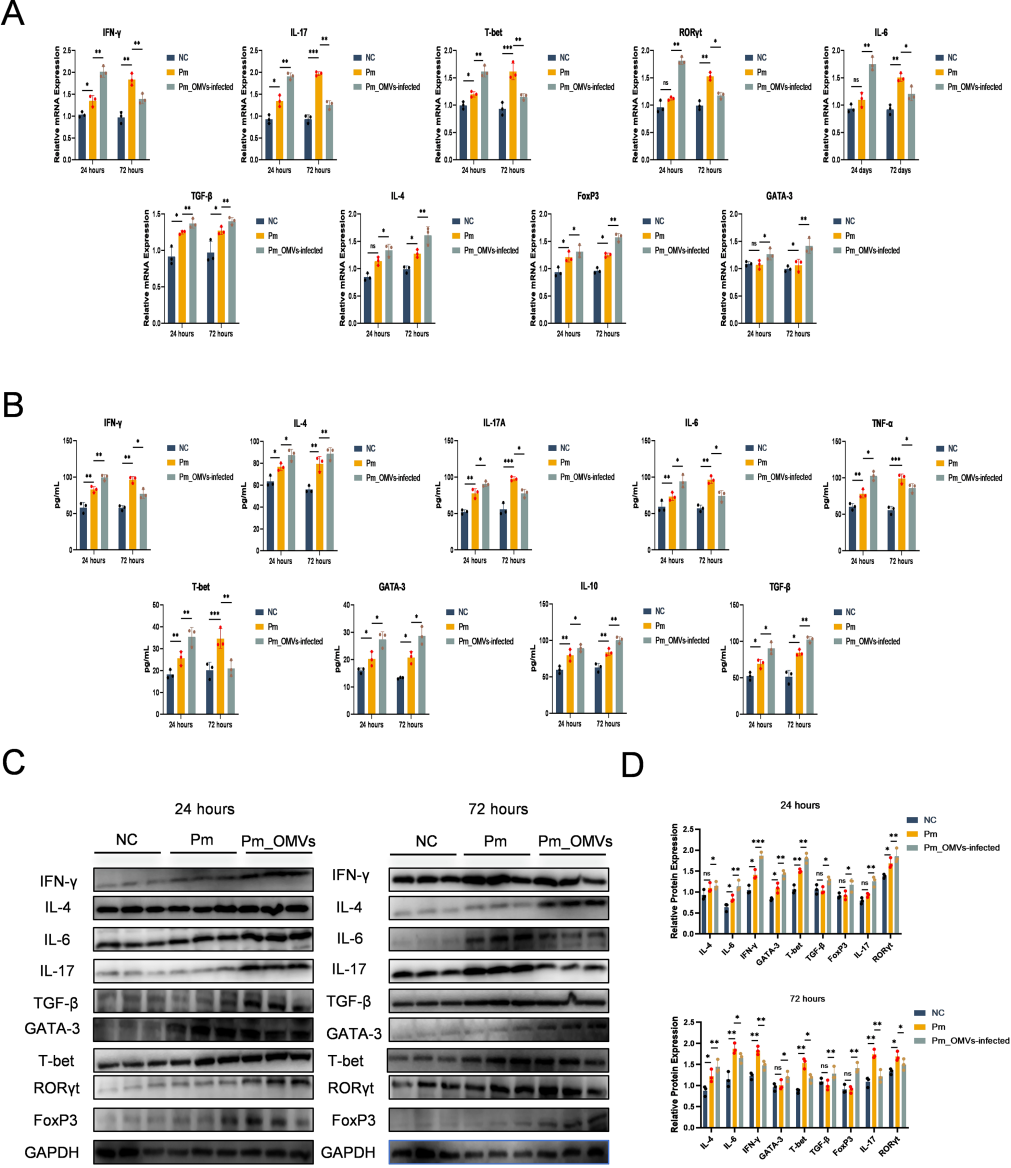
*Pm*_OMVs induces Th cell polarisation to protect *Pm*-infected mice. **(A)** Changes in the expression levels of IFN-γ, IL-4, IL-6, IL-17, TGF-β, GATA-3, T-bet, RORγt, and FoxP3 mRNA in each group (n=3). **(B)** Changes in the secretion levels of cytokines IFN-γ, IL-4, IL-6, IL-17A, TGF-β, GATA-3, T-bet, IL-10, and TNF-a in each group (n=3). **(C, D)** Changes in protein expression levels of IFN-γ, IL-4, IL-6, IL-17, TGF-β, GATA-3, T-bet, RORγt, and FoxP3 in each group (n=3). ^***^P<0.001, ^**^P<0.01, ^*^P<0.05 refer to the level of significance and ns denotes no significance.

To assess whether *Pm*_OMVs confer protection to *Pm*-infected mice through the induction of Th cell differentiation pathways, a *Pm*_OMVs-immunoinfection mouse model was established in this study. As illustrated in **Figure 4A**, qRT-PCR analysis revealed that, relative to the *Pm*-infected group, the expression levels of IFN-γ, IL-4, IL-6, IL-17, TGF-β, GATA-3, T-bet, RORγt, and FoxP3 genes were significantly elevated in the *Pm*_OMVs-infected group at 24 hours post-infection (P < 0.01). However, at 72 hours post-infection, the expression of IL-17, IFN-γ, T-bet, RORγt, and IL-6 genes was markedly reduced in the *Pm*_OMVs-infected group compared to the *Pm* group (P < 0.01), whereas the expression levels of TGF-β, GATA-3, IL-4, and FoxP3 were significantly increased (P < 0.01). Complementary ELISA data (**Figure 4B**) demonstrated a significant augmentation in the secretion of cytokines IFN-γ, IL-4, IL-6, IL-17A, TGF-β, GATA-3, T-bet, IL-10, and TNF-α in the *Pm*_OMVs-infected group relative to the *Pm* group at 24 hours post-infection (P < 0.01). Conversely, at 72 hours post-infection, the secretion of IL-6, IL-17, T-bet, TNF-α, and IFN-γ was significantly diminished (P < 0.01), while levels of TGF-β, GATA-3, IL-10, and IL-4 were significantly elevated in the *Pm*_OMVs-infected group compared to the *Pm* group (P < 0.01). Further validation via Western blot analysis (**Figures 4D, E**) indicated that protein expression levels of IFN-γ, IL-4, IL-6, IL-17, TGF-β, GATA-3, T-bet, RORγt, and FoxP3 were significantly increased in the *Pm*_OMVs-infected group at 24 hours post-infection relative to the *Pm* group (P < 0.01). At 72 hours post-infection, protein levels of IL-17, IFN-γ, T-bet, RORγt, and IL-6 were significantly decreased, whereas TGF-β, GATA-3, IL-4, and FoxP3 protein expression was significantly upregulated in the *Pm*_OMVs-infected group compared to the *Pm* group (P < 0.01).

In summary, at 24 hours post-infection with *Pm* (representing the early stage of infection), the expression of markers associated with T helper (Th) cell differentiation was significantly elevated in the *Pm*_OMVs-infected group compared to the *Pm*-infected group. Notably, markers indicative of Th1 and Th17 cell polarization exhibited significantly higher expression levels than those related to Th2 and regulatory T (Treg) cells, demonstrating that *Pm*_OMVs elicit a robust Th1/Th17 immune response during the initial phase of infection. Conversely, at 72 hours post-infection (the late stage), the *Pm*_OMVs-infected group showed a marked reduction in Th1/Th17 polarization markers relative to the *Pm* group, while markers associated with Th2 and Treg polarization were significantly upregulated. These results indicate that *Pm*_OMVs promote Th2/Treg immune responses during the later stage of infection. Collectively, these findings suggest that *Pm*_OMVs modulate Th cell differentiation throughout the course of *Pm* infection, thereby contributing to its protective immunological effects.

### 3.5 Protective effect of *Pm*_OMVs immunization on *Pm*-infected mice

**Figure 5:**
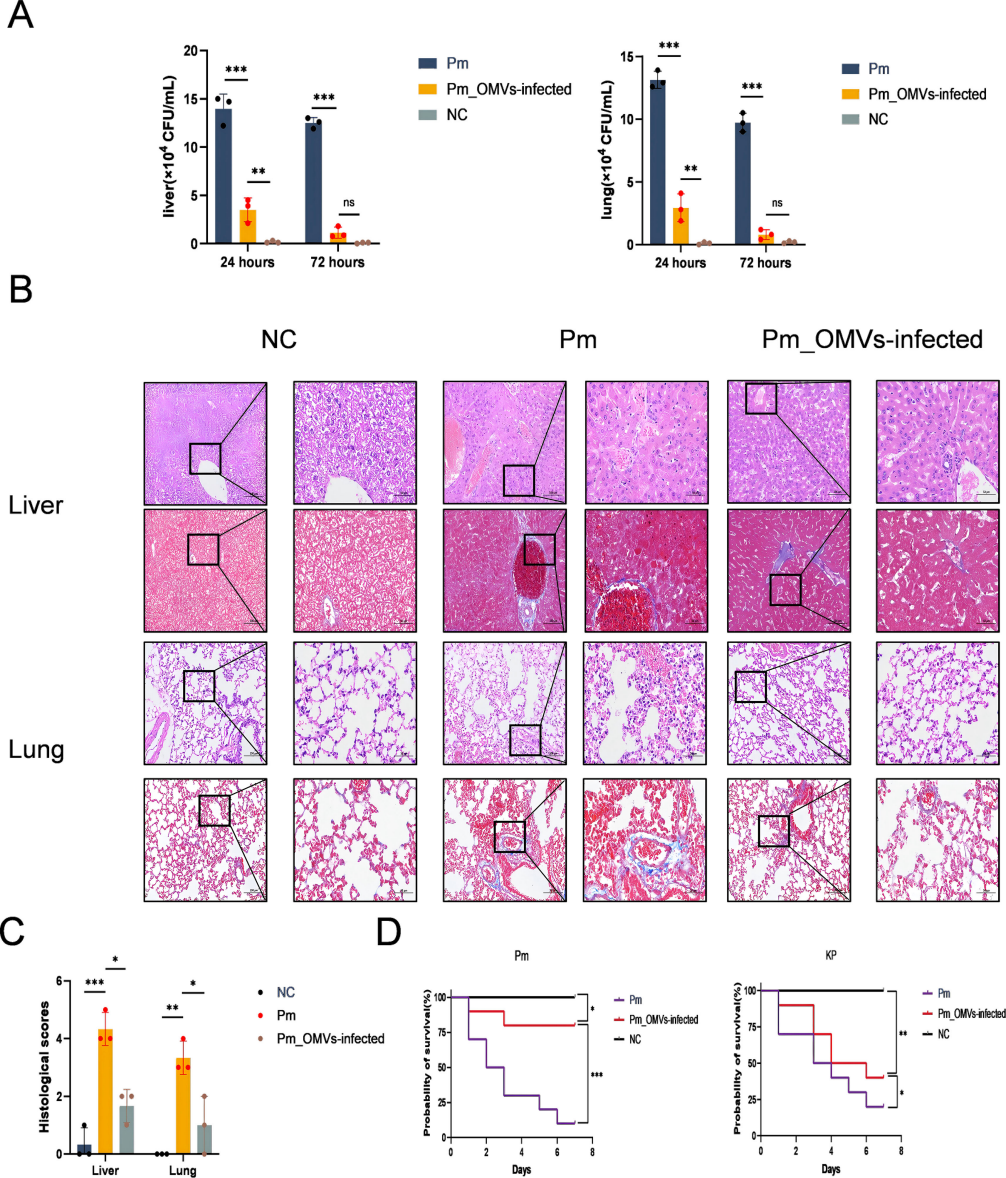
Immunoprotection of *Pm*_OMVs against *Pm*-infected mice. **(A)** Bacterial load of *Pm* group and *Pm*_OMVs-infected group after 24 and 72h of *Pm* infection (n-3). **(B)** HE and Masson staining results of liver and lung of each group (200×, 400×). **(C)** Histological scores of liver and lung pathology in NC group, *Pm* group, and *Pm*_OMVs group (n=3). **(D)** Survival curves of each group at 7 d after *Pm*, *KP* infection (n=10). ***P<0.001, **P<0.01, *P<0.05, refer to the level of significance and ns denotes no significance.

To evaluate the inhibitory effect of *Pm*_OMVs-induced immunity on bacterial proliferation *in vivo*, this study assessed the bacterial load in both the NC, Pm, and the *Pm*_OMVs-infection group at 24 and 72 hours post-*Pm* infection. As illustrated in **Figure 5A**, 24 hours following *Pm* infection, the bacterial load in the liver and lungs of the *Pm*_OMVs-immunized group was significantly reduced compared to that of the *Pm* group (P < 0.001). Furthermore, by 72 hours post-infection, the bacterial load in the *Pm*_OMVs group approached zero, suggesting that the immune response elicited by *Pm*_OMVs effectively inhibited and eradicated the pathogen during the early stages of infection.

To evaluate the immunoprotective efficacy of *Pm*_OMVs in mice challenged with Pm, this study performed comprehensive pathological and histological analyses, including scoring, alongside assessments of physiological and biochemical parameters in critical organs. The results indicated that mice infected with Pm exhibited hepatocellular necrosis and extensive infiltration of inflammatory cells, accompanied by tissue fibrosis. Moreover, disruption of alveolar architecture was observed, characterized by thickening of alveolar walls and evidence of hemorrhage. Conversely, liver and lung tissues from the *Pm*_OMVs-infected group maintained structural integrity, with only minimal inflammatory cell infiltration detected (**Figure 5B**), and demonstrated a significant decrease in histological scores (**Figure 5C**). Additionally, evaluation of physiological and biochemical markers revealed marked reductions in aspartate aminotransferase, blood urea nitrogen, and alanine aminotransferase levels in the *Pm*_OMVs group relative to the Pm group (P < 0.01), indicating that immunization with *Pm*_OMVs effectively preserved hepatic and renal function in Pm-infected mice (**Table 1**).

The immunoprotection rate serves as a critical metric for evaluating immunoprotective efficacy. As illustrated in **Figure 5D**, the survival rate of mice immunized with *Pm*_OMVs and subsequently infected with *Pm* was 80% after seven days of infection. In contrast, the survival rate in the *Pm* group without immunization was only 20%. To further assess the specificity of the immune protection conferred by *Pm*_OMVs, mice immunized with *Pm*_OMVs were challenged with *KP* infection. The findings indicated that the survival rate in the *Pm*_OMVs group following *KP* infection was 40% after seven days, suggesting that *Pm*_OMVs confers only partial, non-specific protection against *KP* infection.

## 4 Discussion

Numerous investigations have demonstrated that OMVs secreted by pathogenic bacteria can modulate the host immune response, thereby providing specific protection in infected murine models and underscoring their potential as valuable immunological agents (24, 25). However, the LPS and Toll-like receptor (TLR) agonists contained within OMVs can provoke immune reactions that may also lead to inflammatory tissue damage (26). Therefore, precise regulation of the immunological dosage of OMVs is essential. In the current study, the immunization dose was systematically optimized through preliminary experiments, resulting in the selection of 80 μg of *Pm*_OMVs for immunization trials. Assessments of histopathological, physiological, and biochemical parameters indicated that administration of *Pm*_OMVs in this study did not cause organ lesions or functional impairments in immunized mice, thereby meeting established criteria for immunological safety. Nonetheless, strict control of endotoxins is essential for the clinical translation of vaccines. Currently, prevalent techniques include density gradient centrifugation, genetic engineering, and detergent washing (27, 28). While these approaches can effectively reduce the endotoxin levels in OMVs, challenges remain in guaranteeing extraction efficiency, maintaining vesicle integrity, and preserving adequate immunogenicity. Addressing these issues will constitute a critical focus of future investigations.

In recent years, multidrug-resistant *Pm* has increasingly emerged within clinical settings, presenting a substantial therapeutic challenge (29). Vaccination remains the most cost-effective strategy for the prevention of infectious diseases. *Pm*_OMVs, characterized by strong immunogenicity, represent a promising antigen candidate for the development of novel vaccines; however, investigations into their immunoprotective efficacy remain limited. In the present study, a *Pm*_OMVs-immunoinfection mouse model was established, revealing that the survival rate of mice with *Pm*_OMVs reached 80%, significantly higher than that of the *Pm* group. Conversely, the survival rate of mice immunized with *Pm*_OMVs and subsequently infected with *KP* was only 40%. These findings indicate that *Pm*_OMVs confer specific protective immunity against *Pm* infection in mice.

Although it has been demonstrated that *Pm*_OMVs provide targeted protection in mice infected with *Pm*, the exact immunoprotective mechanisms involved remain to be fully elucidated. Prior studies have shown that the pertussis OMVs vaccine induces elevated levels of specific antibodies, namely IgG2a and IgG1, which contribute to the suppression of bacterial proliferation and prolongation of survival in infected mice; however, these antibodies did not prevent mortality (30). This phenomenon may be explained by the capacity of these antibodies to activate the complement system or enhance phagocytosis for pathogen clearance, while lacking the ability to neutralize bacterial toxins or achieve complete bacterial eradication. In the present investigation, Specific antibodies, including IgG, IgG1, IgG2a, and IgE, were detected utilizing Pm_OMVs as the substrate antigen. The findings indicated that Pm_OMVs effectively elicited a substantial production of specific antibodies. Nevertheless, this assay could not definitively exclude the contribution of nonspecific immune responses induced by LPS. Consequently, the detection of specific antibodies employing outer membrane proteins such as OmpA or OmpC as substrate antigens may provide a more precise assessment. These antibody responses represent an important aspect of the immune response elicited by *Pm*_OMVs. However, they do not appear to be the primary determinant of its immunogenic efficacy.

Beyond the role of specific antibodies, the contribution of Th cell-mediated cellular immunity warrants significant consideration. Previous studies have demonstrated that mice deficient in IFN-γ exhibit increased susceptibility to bacterial infections relative to wild-type counterparts, underscoring the pivotal role of Th1 cell responses in conferring resistance to bacterial pathogens (31, 32). Furthermore, upon bacterial invasion, Th17 cells facilitate the recruitment of neutrophils to the infection site, thereby curtailing bacterial proliferation and enhancing phagocytic activity to exert bactericidal effects (10, 33). Evidences indicate that OMVs derived from pathogenic Escherichia coli can elicit immune responses predominantly characterized by Th1 and Th17 cell activation, which confer protection in infected murine models (34). Nevertheless, owing to the heterogeneous composition of OMVs, the nature of the immune response they provoke varies across bacterial strains (35). For instance, OMVs originating from Pseudomonas aeruginosa primarily induce humoral immunity and a Th2 cell response, which serves to mitigate excessive inflammatory reactions and protect infected mice (36). In the present study, we established an immune model utilizing *Pm*_OMVs and observed that these vesicles predominantly stimulate Th1 and Th17 cell-mediated immune responses. Consequently, the Th1/Th17 cell response appears to be a critical component of the protective immune mechanism elicited by *Pm*_OMVs.

Furthermore, this study demonstrated that the Th cell response induced by *Pm*_OMVs exerts a robust protective effect within the *Pm* infection model. *Pm* possesses the capacity to form biofilms in complex environments, which impedes phagocytic uptake and diminishes the bactericidal activity of the complement system (37, 38). This immune evasion strategy delays rapid recognition by the host immune system and the subsequent initiation of an effective immune response to eliminate the pathogen (39). Notably, at 24 hours post-*Pm* infection, the expression levels of Th1 and Th17 cell differentiation markers were significantly elevated in the *Pm*_OMVs-infected group relative to the *Pm* group, concomitant with a marked reduction in bacterial burden within lung and liver tissues. These findings indicate that mice immunized with *Pm*_OMVs can promptly recognize *Pm* antigenic components and mount an immune response capable of inhibiting or eradicating the pathogen during the early phase of infection. Moreover, in the prevention and treatment of bacterial diseases, it is essential to secure a favorable prognosis in conjunction with the thorough eradication of the pathogens (40). Conventional antibiotic treatment of multidrug-resistant Helicobacter pylori can be effective but is frequently associated with chronic or latent infections, resulting in recurrent disease and potential organ damage (41). And single-antigen vaccines that elicit either T- or B-cell responses may confer prophylactic benefits; however, the excessive inflammatory responses they provoke can lead to immune-mediated tissue injury (42). In the present study, at 72 hours post-*Pm* infection, bacterial loads in the lungs and liver of the *Pm*_OMVs-infected group approached zero, accompanied by significantly elevated expression of Th2 and Treg cell differentiation markers compared to the *Pm* group. Although hyperactivation of Th2 and Treg cells can suppress immune responses and potentially hinder complete pathogen clearance (43), histopathological analysis revealed that mice in the *Pm*_OMVs-infected group exhibited minimal inflammatory cell infiltration, absence of irreversible fibrosis or necrosis, and significant improvements in physiological and biochemical parameters relative to the *Pm* group. These results suggest that Th2 and Treg responses may contribute to favorable clinical outcomes by modulating immune activity and promoting tissue repair during the resolution phase following pathogen clearance.

In summary, the *Pm*_OMVs employed in this investigation effectively elicited both B-cell and T-cell immune responses while maintaining immunological safety. The adaptive immunity triggered by *Pm*_OMVs in *Pm*-infected mice conferred specific protection against infection, enhanced survival rates, and contributed to favorable prognostic outcomes. These findings indicate that *Pm*_OMVs represents a promising antigen candidate for the development of *Pm* vaccines.

## Data Availability

The original contributions presented in the study are included in the article/**Supplementary Material**. Further inquiries can be directed to the corresponding author/s.
