# Supplementary figures and images for: Immunization with outer membrane vesicles drived Proteus mirabilis protects mice against bacteria-induced lethality
Source: Front Immunol. 2025 Nov 11;16:1688837. doi: 10.3389/fimmu.2025.1688837 (PMC12644052; doi:10.3389/fimmu.2025.1688837)

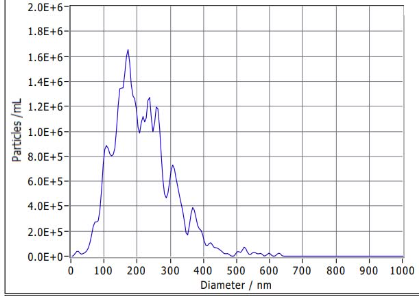

Supplement: Supplementary file 3 [file DataSheet3.zip › supply NTA data/NTA data.png]

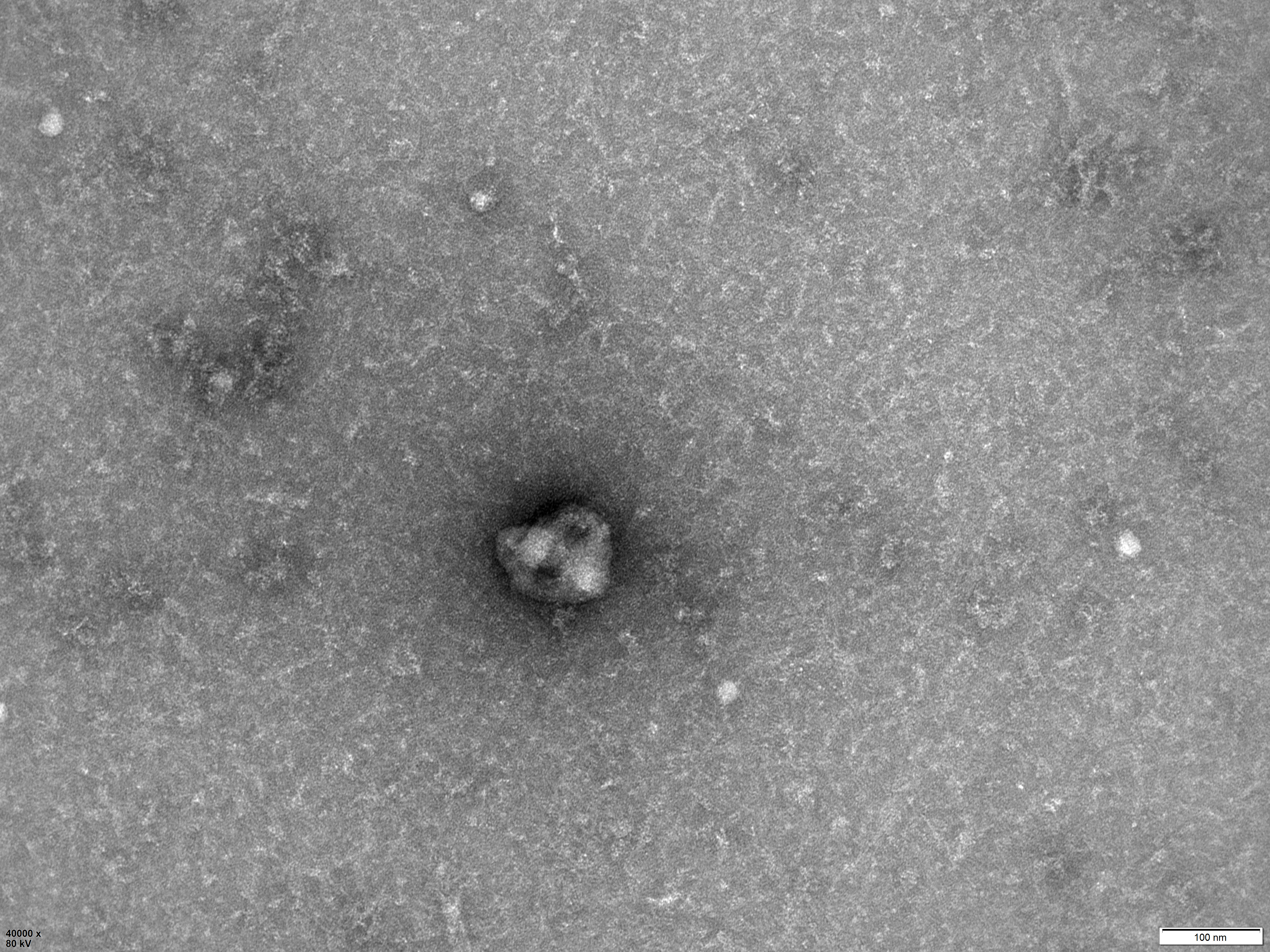

Supplement: Supplementary file 4 [file Image1.png]

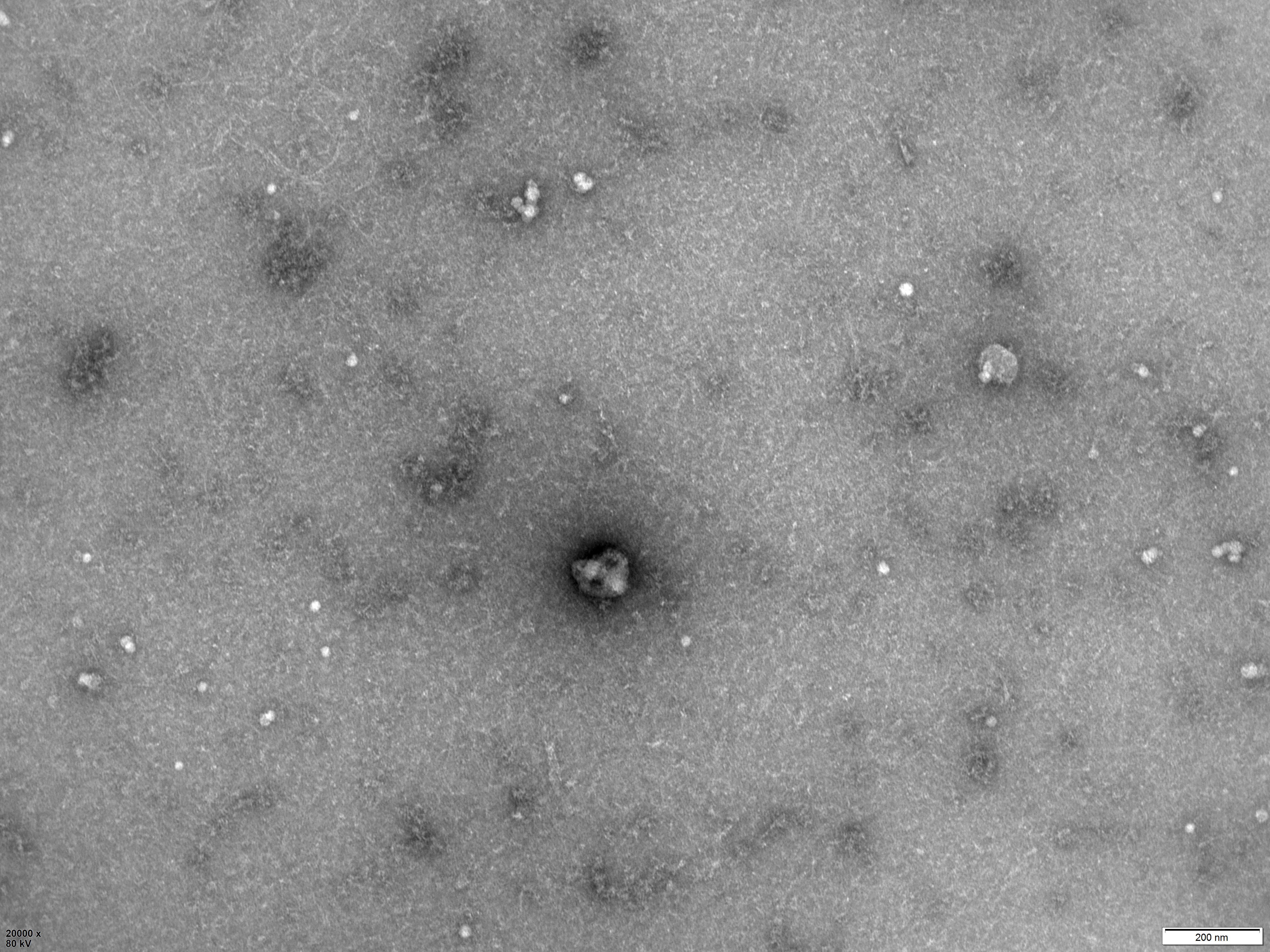

Supplement: Supplementary file 5 [file Image2.png]

## Slide 1
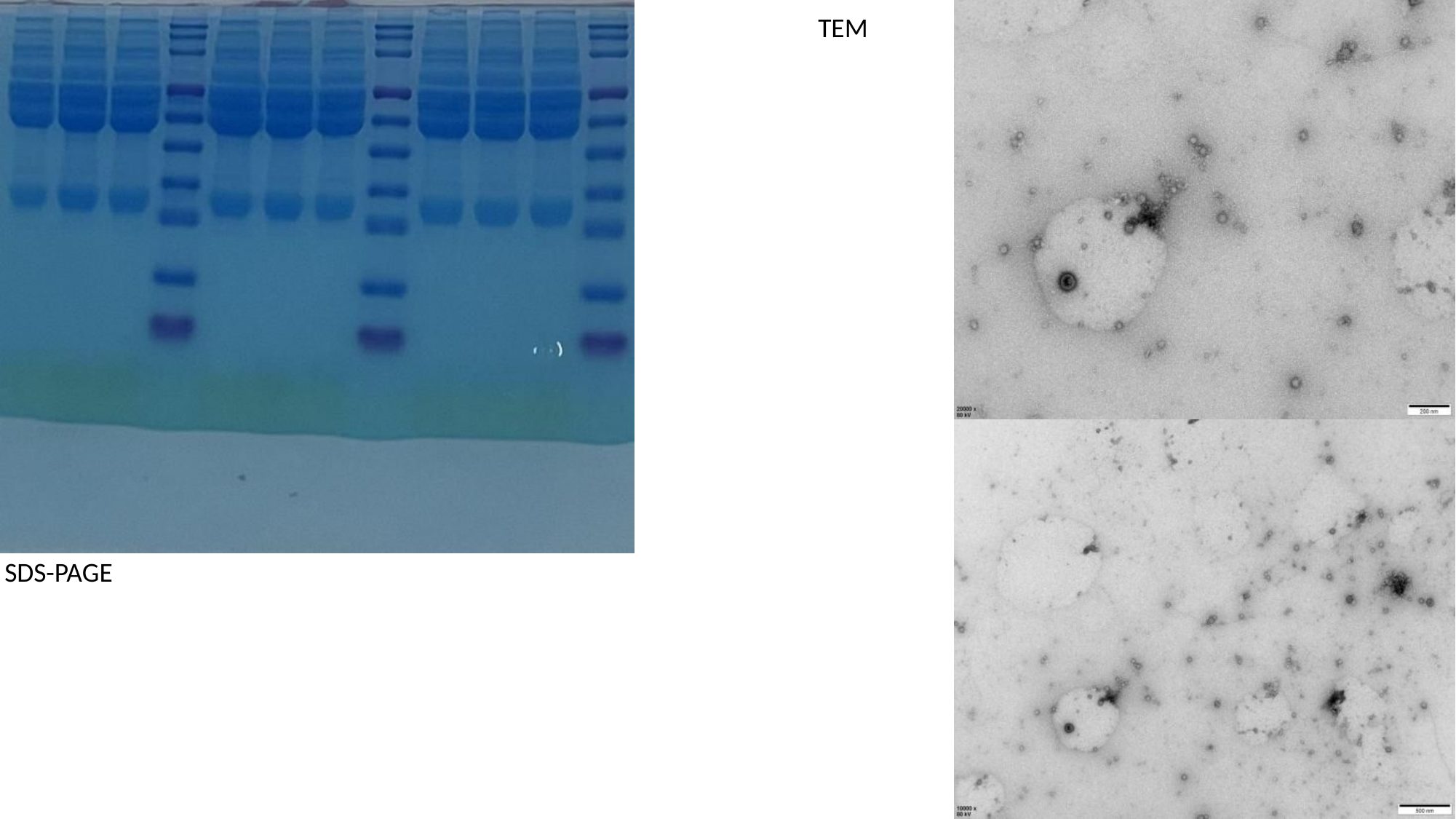

TEM
SDS-PAGE

Supplement: Supplementary file 7 [file Presentation1.pptx]
