# Supplementary material for: Immunization with outer membrane vesicles drived Proteus mirabilis protects mice against bacteria-induced lethality
Source: Front Immunol. 2025 Nov 11;16:1688837. doi: 10.3389/fimmu.2025.1688837 (PMC12644052; doi:10.3389/fimmu.2025.1688837)
Supplement: Supplementary file 8 [file Presentation2.pptx]

## Slide 1
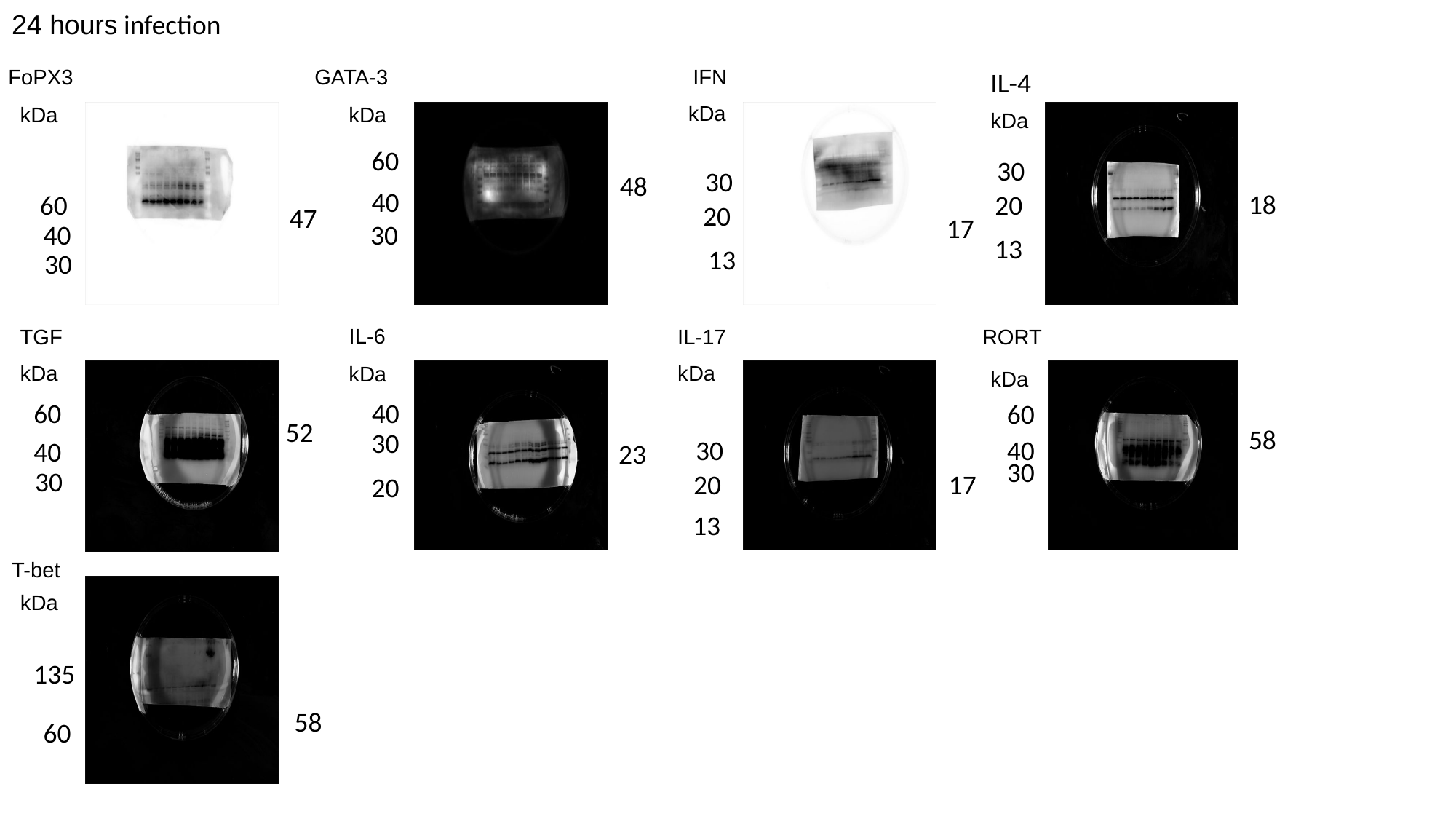

24 hours infection
FoPX3
GATA-3
IFN
IL-4
kDa
kDa
kDa
kDa
60
30
30
48
40
18
60
20
20
47
17
40
30
13
13
30
IL-6
TGF
IL-17
RORT
kDa
kDa
kDa
kDa
60
40
60
52
58
30
30
40
40
23
30
30
20
17
20
13
T-bet
kDa
135
58
60

## Slide 2
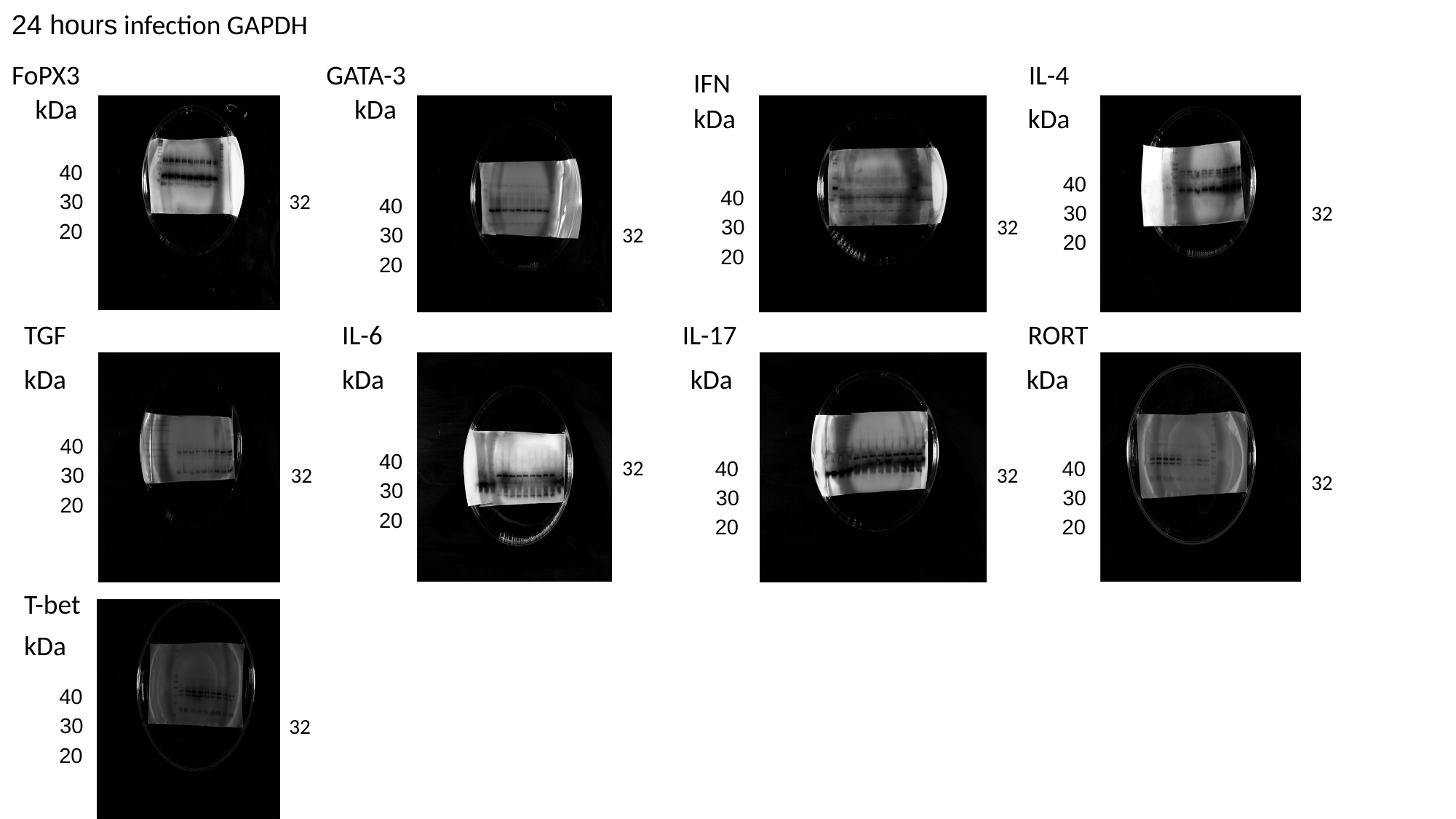

24 hours infection GAPDH
FoPX3
GATA-3
IL-4
IFN
kDa
kDa
kDa
kDa
40
40
40
30
32
40
30
32
30
32
20
30
32
20
20
20
IL-17
RORT
TGF
IL-6
kDa
kDa
kDa
kDa
40
40
32
40
40
30
32
32
32
30
30
30
20
20
20
20
T-bet
kDa
40
30
32
20

## Slide 3
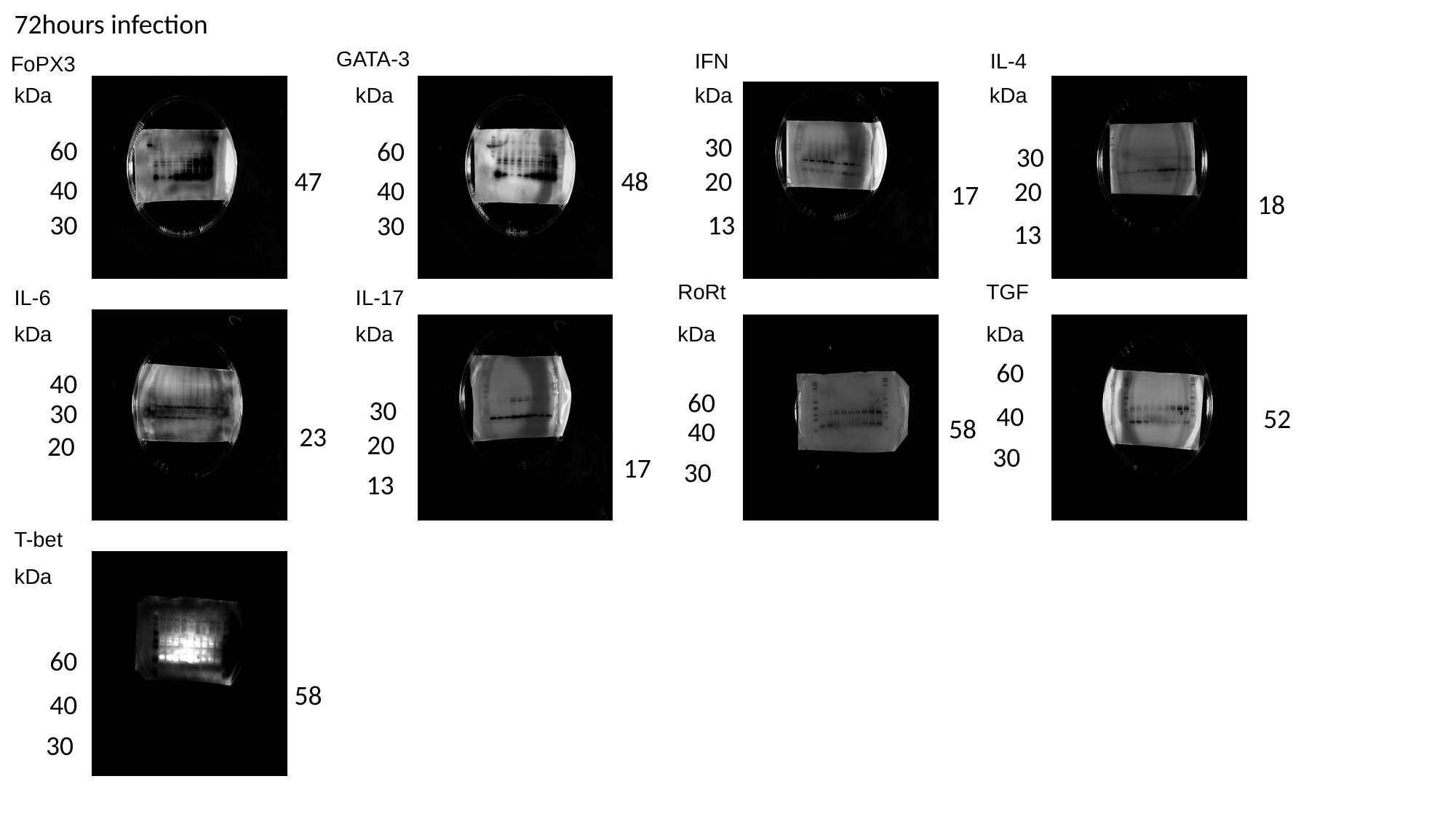

72hours infection
GATA-3
IFN
IL-4
FoPX3
kDa
kDa
kDa
kDa
30
60
60
30
47
20
48
40
40
20
17
18
30
13
30
13
RoRt
TGF
IL-17
IL-6
kDa
kDa
kDa
kDa
60
40
60
30
30
40
52
58
40
23
20
20
30
17
30
13
T-bet
kDa
60
58
40
30

## Slide 4
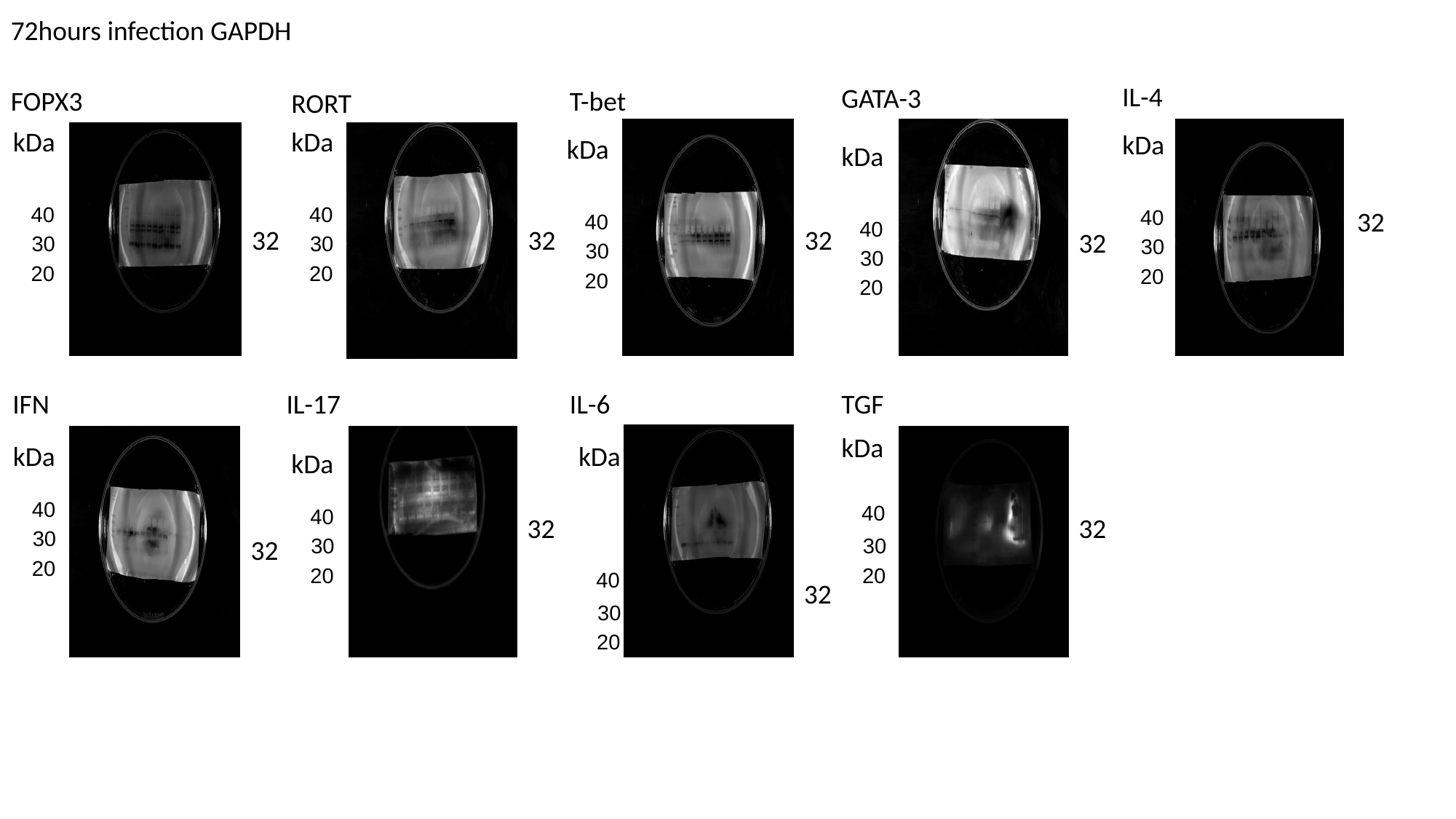

72hours infection GAPDH
IL-4
GATA-3
FOPX3
T-bet
RORT
kDa
kDa
kDa
kDa
kDa
40
40
40
32
40
40
32
32
32
32
30
30
30
30
30
20
20
20
20
20
IFN
IL-17
IL-6
TGF
kDa
kDa
kDa
kDa
40
40
40
32
32
30
30
30
32
20
20
20
40
32
30
20

## Slide 5
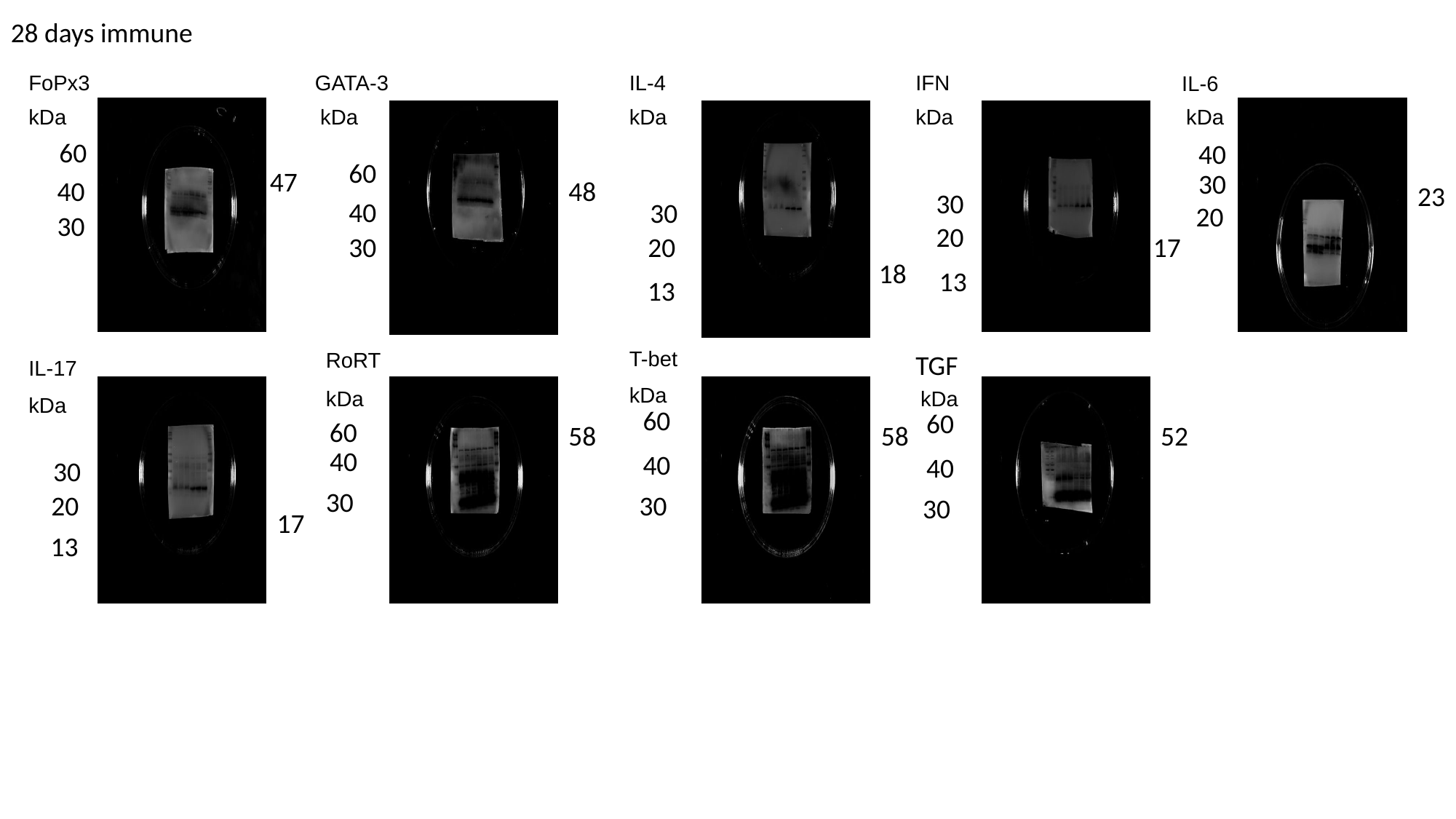

28 days immune
FoPx3
GATA-3
IL-4
IFN
IL-6
kDa
kDa
kDa
kDa
kDa
60
40
60
47
30
40
48
23
30
40
30
20
30
20
30
17
20
18
13
13
T-bet
RoRT
TGF
IL-17
kDa
kDa
kDa
kDa
60
60
60
58
58
52
40
40
40
30
30
20
30
30
17
13

## Slide 6
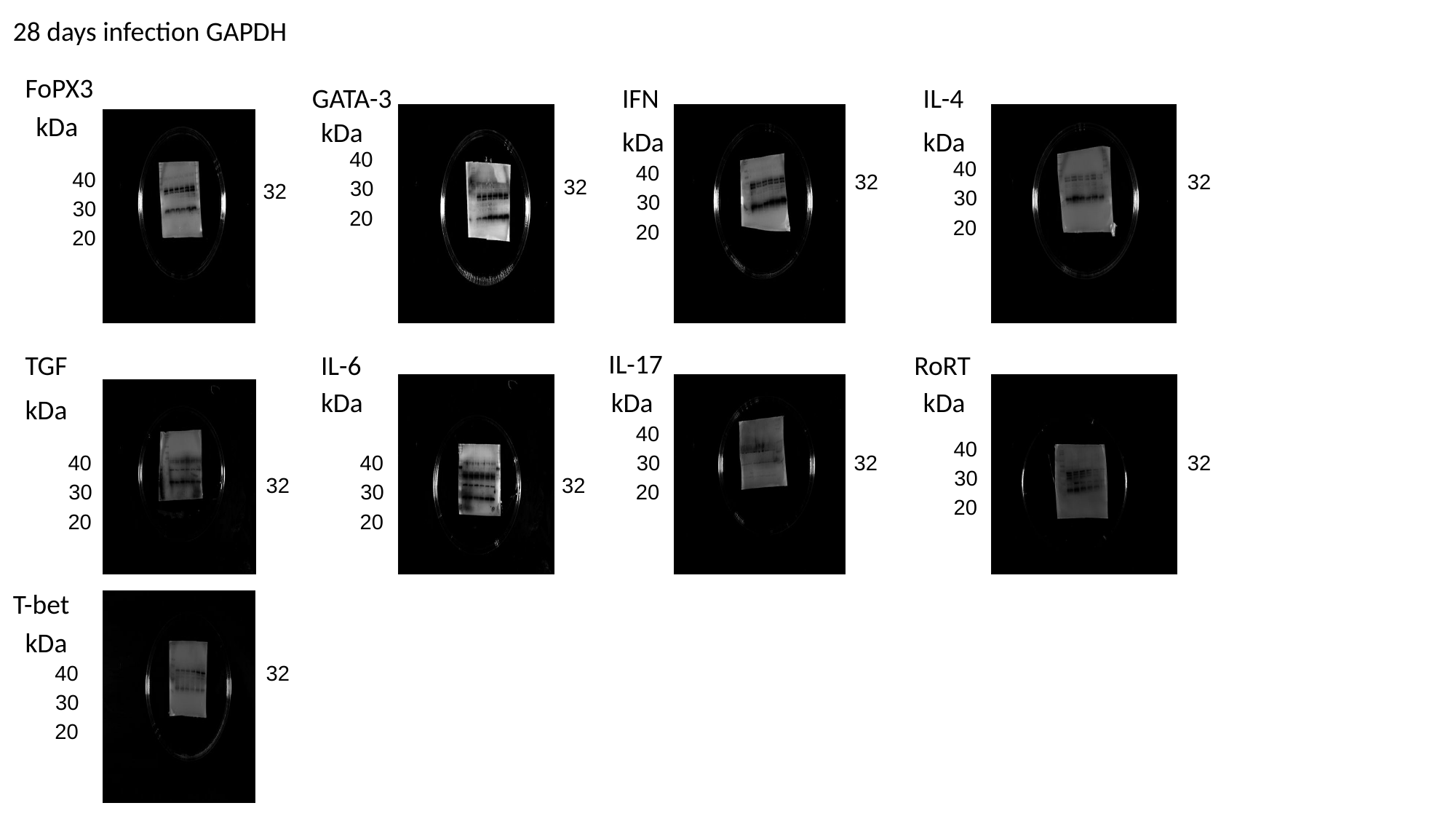

28 days infection GAPDH
FoPX3
IFN
GATA-3
IL-4
kDa
kDa
kDa
kDa
40
40
40
40
32
32
32
30
32
30
30
30
20
20
20
20
IL-17
TGF
IL-6
RoRT
kDa
kDa
kDa
kDa
40
40
30
32
32
40
40
30
32
32
30
30
20
20
20
20
T-bet
kDa
40
32
30
20

## Slide 7
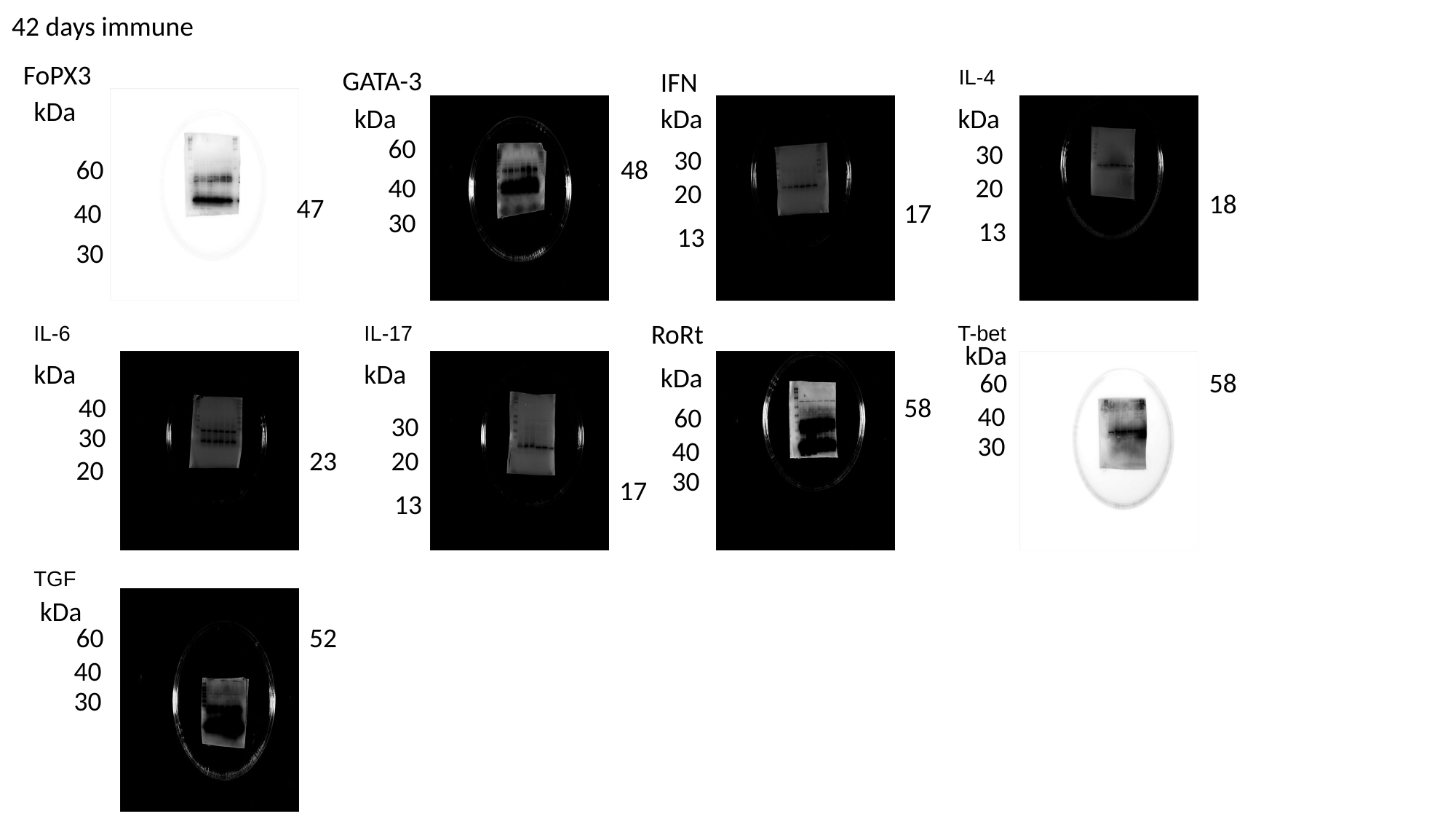

42 days immune
FoPX3
GATA-3
IL-4
IFN
kDa
kDa
kDa
kDa
60
30
30
60
48
40
20
20
18
47
40
17
30
13
13
30
RoRt
IL-6
IL-17
T-bet
kDa
kDa
kDa
kDa
58
60
40
58
40
60
30
30
30
40
23
20
20
30
17
13
TGF
kDa
60
52
40
30

## Slide 8
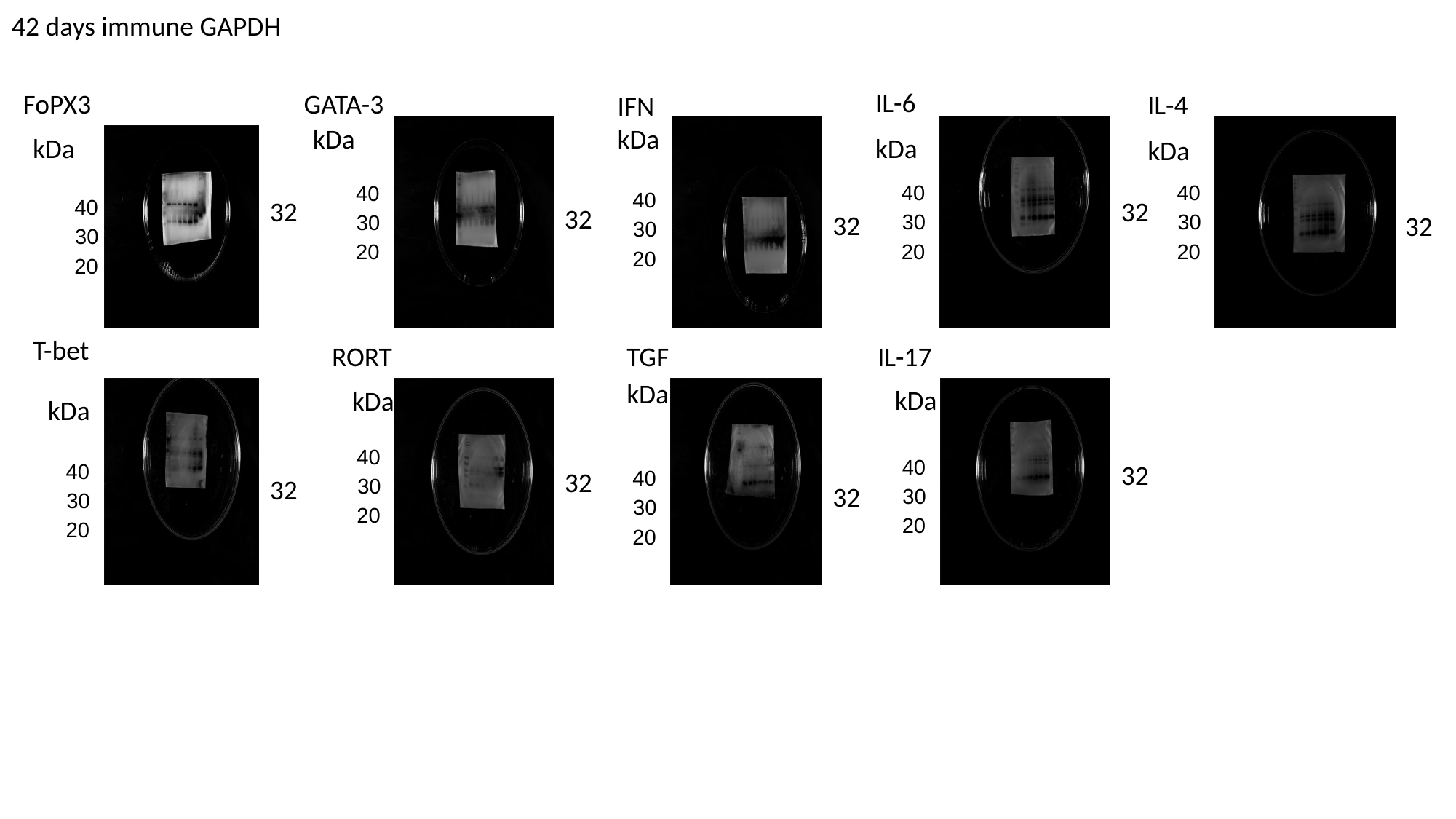

42 days immune GAPDH
IL-6
FoPX3
GATA-3
IL-4
IFN
kDa
kDa
kDa
kDa
kDa
40
40
40
40
40
32
32
32
32
30
30
30
32
30
30
20
20
20
20
20
T-bet
IL-17
RORT
TGF
kDa
kDa
kDa
kDa
40
40
32
40
40
32
32
30
32
30
30
30
20
20
20
20
